# Managing uncertainty of expert’s assessment in FMEA with the belief divergence measure

**DOI:** 10.1038/s41598-022-10828-2

**Published:** 2022-04-26

**Authors:** Yiyi Liu, Yongchuan Tang

**Affiliations:** School of Big Data and Software Engineering, Chongqing University, Chongqing, 401331 China

**Keywords:** Electrical and electronic engineering, Computer science

## Abstract

Failure mode and effects analysis (FMEA) is an effective model that identifies the potential risk in the management process. In FMEA, the priority of the failure mode is determined by the risk priority number. There is enormous uncertainty and ambiguity in the traditional FMEA because of the divergence between expert assessments. To address the uncertainty of expert assessments, this work proposes an improved method based on the belief divergence measure. This method uses the belief divergence measure to calculate the average divergence of expert assessments, which is regarded as the reciprocal of the average support of assessments. Then convert the relative support among different experts into the relative weight of the experts. In this way, we will obtain a result with higher reliability. Finally, two practical cases are used to verify the feasibility and effectiveness of this method. The method can be used effectively in practical applications.

## Introduction

Risk assessment and prevention have drawn more and more attention in modern management. Risk represents the probability of an adverse event which will breach security and pose a threat. Assessments of risk are largely dependent on an analysis of the uncertainty. Failure mode and effects analysis (FMEA), a risk assessment method widely used in engineering and management^1^, was first proposed by the Department of Defense, USA in 1949^2^ and used to solve quality and reliability problems in military products. FMEA has been gradually applied to all walks of life, including aerospace^3^, automobile manufacturing^4^, the medical field^5^, food safety^6^, and supplier selection^7^. The main purpose of FMEA is to identify potential failure modes and assess their causes and influences^8^. The core parameter of FMEA is risk priority number (RPN)^9^, which is the product of three risk factors, which are the occurrence (O), severity (S), and detection (D) of a failure mode. The failure modes are ranked according to their RPN, and the failure mode with the highest RPN has the higher priority.

The traditional FMEA model can be roughly described as the following steps. (1) Identifying all failure modes in the target system. (2) Assessing the risk factors of these failure modes by experts. (3) Calculating the RPN value of failure modes according to the result of assessments. (4) Ranking the failure modes on the basis of RPN value. However, in practice, there is a great deal of uncertainty in assessing potential risks in systems with the traditional FMEA model, often yielding imprecise results. Because it is difficult to reach an agreement on the assessment of failure mode by different experts^10^, coupled with the inaccurate cognition of the real problem by experts, the assessment of risk is inaccurate and uncertain^11^. For example, if a very authoritative expert gives an assessment of a failure mode is (5,6,7) (assuming that his assessment is very close to the truth), the RPN value is 210. And another expert gives an assessment is (3,1,4). The RPN value is 12. Obviously, due to the second expert’s subjective opinion or incomplete understanding of the problem, their assessment has great ambiguity and uncertainty. The average RPN value is 111. It is very different from the real situation. In addition, the traditional FMEA has some defects^12,13^. First, the traditional FMEA model ignored the relative importance between the three risk factors named O, S, and D. Different risk factors should have different weights, so there is no way to unify the weights of the three risk factors. Second, the traditional FMEA model divides ratings of O, S, and D into non-linear scales of grades [1, 2, 3, ..., 10]. It will eventually produce many repeated and intermittent values that will affect the ability of the management personnel to make effective decisions. Third, there are some subjective assumptions about the assessments of the failure mode by experts. Enough attention should be given to the weighting of each expert.

For the above problems, some existing studies propose many methods to deal with the uncertainty in risk assessments by adopting existed theories such as fuzzy sets theory^14,15^, Dempster-Shafer evidence theory^16^, evidence reasoning^17^, prospect theory^18^, D-number theory^19^, Z-number theory^20^, R-number theory^21^, fairness-oriented consensus approach^22^, grey relation analysis method^23^, and best-worst method^24^. Among them, Liu et al. propose a method combining the fuzzy theory and technique for order preference by similarity to ideal solution (TOPSIS)^25^, which achieves the calculation of weights of expert decisions based on similarity. Wang et al. capture the experts’ diverse assessments on the risk of failure modes and the weights of risk factors by interval two-tuple linguistic variables and develop a ranking method for failure modes based on the regret theory and TODIM^26^. In^27^, the authors use the ambiguity measure(AM) to quantify the degree of uncertainty assessed by each expert for each risk item. An AM-based weighting method for weighted risk priority number is proposed in^28^. A FMEA method based on rough set and interval probability theories is proposed in^29^, which converts the assessment values of risk factors into interval numbers, and the interval exponential RPN is proposed to overcome the discontinuity problem of traditional RPN values. In^30^, the authors propose a FMEA method based on Deng entropy under the Dempster-Shafer evidence theory framework, where the uncertainty of expert assessments is measured by Deng entropy and converted into the relative weights of experts and weights of risk factors. In addition to the above studies, some researchers have done some studies based on similarity measure in FMEA . In^31^, Zhou et al. use the Similarity Measure Value Method (SMVM) to model the failure modes and their correlations. This method gains similarity among assessments based on the concept of medium curve and fuzzy number. Pang et al. propose a method to weight the experts based on the similarity of their assessments, which is calculated by fuzzy Euclidean distance^32^. Furthermore, Jin et al.’s research introduce the Dice similarity and the Jaccard similarity^33^. However, little research is conducted to improve FMEA from the standpoint of divergence measure, despite the fact that divergence measure and similarity measure share some characteristics, while Song and Wang use the form of “$1-D(A,B)$” (*D*(*A*, *B*) represents the divergence of evidence) to measure the similarity^34^. Most previous researches have improved the FMEA in view of the process of assessment. Those methods are able to effectively model the experts’ assessments as accurate data and deal with them with some appropriate methods. But for the data that has been modeled, it is necessary to measure the uncertainty among them by some methods, such as the divergence measure. Due to the fact that there is little research which combines the divergence measure and FMEA, the effectiveness of the method that introduces divergence measure into FMEA is necessary to verify. It’s also the motivation of this paper.

Because of the influence of subjective opinion and historical experience, expert assessments are often inaccurate. The uncertainty among the assessments by different experts needs to be measured by some appropriate methods. Processing data with imprecise information can be done using the Dempster-Shafer evidence theory^35,36^. In Dempster-Shafer evidence theory, how to measure the divergence and conflicts between the evidence remains an open issue^37^. There are many uncertainty measurement methods^38^, such as ambiguity measure^39^, total uncertainty measure^40^, divergence measure^41^, the correlation coefficient^42^, and the fractal-based belief entropy^43^. Recently, Xiao^44^ proposed the belief divergence measure (BJS) on the basis of the Jensen-Shannon divergence measure^45^. By replacing the probability assignment function with the mass function, BJS is able to effectively measure the divergence between different pieces of evidence. Therefore, this work propose an expert assessment uncertainty analysis method based on BJS.

The new method models the belief structure of expert assessment results, calculate the divergence among BPAS with BJS, and construct the divergence degree matrix. Since the divergence degree and the support degree of assessments are opposite concepts, the divergence degree of other BPAS to the current BPA is regarded as the reciprocal of the support degree. This theory is used to convert the average divergence degree into the average support degree, which is used to represent the weight of experts. By bringing the weight of experts into the calculation of RPN, a more accurate analysis of expert assessments will be obtained and the risk of the system will be reduced. Compared with other improved methods, BJS calculates the reliability by combining all the evidence rather than calculating the credibility of each piece of evidence in isolation, so the results calculated in this way have higher reliability. In addition, the method considers the relative importance of different experts, reduces the uncertainty caused by divergence that is produced by the subjectivity of different experts, and is more in line with the actual situation.

This paper’s contribution is that the new method proposed solutions in view of the traditional FMEA defects, in this way, provide a new idea to improve the FMEA method. In addition, this paper provides some new theoretical support for the research combining divergence and FMEA. The rest of this work is organized as follows: in Preliminaries" section reviews the theoretical basis of this work. In "FMEA method based on belief divergence measure" section, aiming at FMEA, an expert assessment uncertainty measurement method based on the belief divergence measure is proposed. Then, an actual case is used to verify the application of this method in "Applications and discussion" section. Finally, "Conclusion" section summarizes the content of this work.

## Preliminaries

### Dempster-Shafer evidence theory

The D-S evidence theory (DST) is a very effective tool to process the data with uncertainty. From data modeling to uncertainty measurement and data fusion, every step has useful methods to finish. Research on the DST has made great progress in recent years. Accordingly, the FMEA method in DST has great advantages. The DST was first proposed by Dempster in 1967 and further developed by Shafer^46,47^. DST is a generalization of Bayesian subjective probability theory and also an extension of classical probability theory. As a mathematical framework for representing uncertainty, DST combines the degree of belief from independent evidence items. DST is defined as below:

Supposing $\Omega$ is a fixed, exhaustive set of mutually exclusive events whose probability of occurrence does not interfere with each other. $\Omega$ is expressed by the following formula:

1 $$\begin{aligned} \Omega = \lbrace H_1, H_2,H_3,\dots ,H_n \rbrace \end{aligned}$$

where $\Omega$ is called the frame of discernment, and the set of all subsets of $\Omega$(such as formula (2)) is called the power set of $\Omega$, which is recorded as $2^\Omega$.

2 $$\begin{aligned} 2^\Omega = \lbrace \varnothing ,\lbrace H_1\rbrace ,\lbrace H_2\rbrace ,\dots ,\lbrace H_n\rbrace ,\lbrace H_1, H_2\rbrace ,\lbrace H_1, H_2,\dots ,H_n\rbrace \rbrace \end{aligned}$$

where $\varnothing$ is an empty set, and the elements in $2^\Omega$ are called propositions.

The mass function, also known as basic probability assignment (BPA), represents the mapping relationship between an element in $2^\Omega$ and interval [0,1]. It is defined as follows:

3 $$\begin{aligned} m:2^\Omega \rightarrow \left[ 0,1\right] \end{aligned}$$

Mass function also satisfy the condition as follows:

4 $$\begin{aligned} m(\varnothing )=0,\sum _{A\subset \Omega }m(A)=1 \end{aligned}$$

For a focus element A of $\Omega$, its Belief function bel (A) is defined as follows:

5 $$\begin{aligned} Bel(A)=\sum _{B\subseteq A}m(B) \end{aligned}$$

The plausibility function pl (A) of A is defined as follows:

6 $$\begin{aligned} pl(A)=\sum _{A\cap B=\varnothing }m(B) \end{aligned}$$

The bel(A) is the lower bound function of proposition A, and the pl(A) is the upper bound function of proposition A.

Assuming that $m_1$ and $m_2$ are two BPAS under the frame of discernment $\Omega$, B and C are the focus elements of $m_1$ and $m_2$, respectively. By using the Dempster’s combination rule, the two groups of BPAS are fused to obtain a new set of probabilities. Dempster’s combination rule is defined as follows:

7 $$\begin{aligned} m(A)=(m_1 \oplus m_2)(A)=\frac{1}{1-k}\sum _{B\cap C=A} m_1(B)m_2(C) \end{aligned}$$

where k represents the degree of conflict between two evidence bodies, which is called the conflict coefficient, k is defined as follows:

8 $$\begin{aligned} k=\sum _{B\cap C=\varnothing }m_1(B)m_2(C) \end{aligned}$$

### FMEA

FMAE is a management tool for system reliability with a highly structured approach that provides a set of effective technologies for risk assessment and prevention^11,48^, and has been widely used in product quality monitoring, decision-making, other fields. FMEA mainly relies on experts to assess different failure modes so as to determine the priority of each failure mode. Those failure modes with a high RPN value often get focused attention to reduce the risk of the system effectively. The calculation of RPN is an important step in FMEA, and the definition of RPN is as follows:

The RPN consists of the probability of failure occurrence (O), the severity of failure occurrence (S), and the probability of failure being detected (D). The traditional RPN model multiplies the three risk factors (O, S, and D) to obtain the RPN value, as shown in formula 9:

9 $$\begin{aligned} RPN=O\times S\times D \end{aligned}$$

In tradition, the grades of O, S, and D are often divided into 10 levels, in which each level of assessment is given different explanations. The assessment level for O is shown in Table 1, and the assessment levels for S and D can be found in^49^.

**Table 1:** Classification of failure mode occurrence probability.

Level	Possibility of failure	Probability range of occurrence
10	Extremely high	$\ge$ 1/2
9	Very high	1/3
8	Slightly high	1/8
7	High	1/20
6	Middle high	1/80
5	Middle	1/400
4	Relatively low	1/2000
3	Low	1/15000
2	Slightly low	1/150000
1	Hardly occurs	1/1500000

### Divergence measure

The divergence measure can effectively measure the divergence and conflict between evidence. The divergence, like the similarity, measures the conflict from a distance perspective, but the divergence and similarity are diametrically opposed concepts.There are many existing divergence measurements, summarized below.

For two probability distributions $A={a_1,a_2,\dots ,a_n}$ and $B={b_1,b_2,\dots ,b_n}$. The JS divergence measure is denoted as^45^:

10 $$\begin{aligned} JS(A,B)=\frac{1}{2}\left[ \sqrt{\sum _iA_i\log \frac{A_i}{\frac{1}{2}A_i+\frac{1}{2}B_i}}+\sqrt{\sum _iB_i\log \frac{B_i}{\frac{1}{2}A_i+\frac{1}{2}B_i}}\right] \end{aligned}$$

The BJS divergence measure was proposed by Xiao based on the JS divergence measure. Supposing that there are two BPAS, $m_1$ and $m_2$, the BJS divergence measure between them is denoted as^44^:

11 $$\begin{aligned} BJS(m_1,m_2)=\frac{1}{2}\left[ S\left( m_1,\frac{ m_1 + m_2}{2}\right) + S\left( m_2,\frac{ m_1 + m_2}{2}\right) \right] \end{aligned}$$

where,

$$\begin{aligned} S(m_1,m_2) = \sum _im_1(A_1)\log \frac{m_1(A_i)}{m_2(A_i)} \end{aligned}$$

BJS is also defined as the following formula:

12 $$\begin{aligned} \begin{aligned} BJS(m_1,m_2)&= H(\frac{m_1 + m_2}{2}) - \frac{1}{2}H(m_1) - \frac{1}{2}H(m_2)\\ {}&= \frac{1}{2}\left[ \sum _im_1(A_i)\log (\frac{2m_1(A_i)}{m_1(A_i)+m_2(A_i)}) + \sum _im_2(A_i)\log (\frac{2m_2(A_i)}{m_1(A_i)+m_2(A_i)})\right] \end{aligned} \end{aligned}$$

where, H($m_j$) represents Shannon entropy, and H ($m_j$) is defined as:

13 $$\begin{aligned} H(m_j) = -\sum _im_j(A_i)\log m_j(A_i) \end{aligned}$$

The Reinforced belief divergence measure (RB divergence measure) was proposed by Xiao in 2019. It mainly measures the divergence among belief functions. For two belief functions in the frame of discernment, m1 and m2, the RB divergence measure is denoted as^50^:

14 $$\begin{aligned} RB(m_1,m_2)=\sqrt{\frac{\left| B(m_1,m_1)+B(m_2,m_2)-2B(m_1,m_2)\right| }{2}} \end{aligned}$$

where

15 $$\begin{aligned} \begin{aligned} B(m_1,m_2)&=\sum _{i=1}^{2^k}\sum _{j=1}^{2^k}m_1(A_i)\log \frac{m_1(A_i)}{\frac{1}{2}m_1(A_i)+\frac{1}{2}m_2(A_j)}\frac{\left| A_i\cap A_j\right| }{|A_j|}\\ &\quad+\sum _{i=1}^{2^k}\sum _{j=1}^{2^k}m_2(A_i)\log \frac{m_2(A_i)}{\frac{1}{2}m_1(A_i)+\frac{1}{2}m_2(A_j)}\frac{\left| A_i\cap A_j\right| }{|A_i|} \end{aligned} \end{aligned}$$

The divergence measure proposed by Wang et al. between m1 and m2 is denoted as^51^:

16 $$\begin{aligned} \begin{aligned} D(m_1,m_2)&=\frac{1}{2}\sum _{\theta _i\subset \Theta }PBl_{m_1}(\theta _i)\log \frac{PBl_{m_1}(\theta _i)}{\frac{1}{2}PBl_{m_1}(\theta _i)+PBl_{m_2}(\theta _i)} \\&\quad + \frac{1}{2}\sum _{\theta _i\subset \Theta }PBl_{m_2}(\theta _i)\log \frac{PBl_{m_2}(\theta _i)}{\frac{1}{2}PBl_{m_1}(\theta _i)+PBl_{m_2}(\theta _i)} \end{aligned} \end{aligned}$$

Compared with Wang et al. divergence, the BJS represents the divergence directly from the view of entropy without calculating the pl function. As for RB divergence, most assessments in FMEA are regarded as propositions with a single element, so the RB divergence will be complex and inefficient in FMEA. The BJS is based on the JS divergence measure and is the extent of the JS divergence measure. BJS is widely used in belief functions. When all the hypothesis of belief functions are assigned to a single element, the BBA will transform into probability. At this time, the BJS will degenerate into JS^44^.

## FMEA method based on belief divergence measure

This work proposed a method for calculating RPN value based on the divergence measure, which uses BJS under the framework of Dempster-Shafer evidence theory to measure the divergence between evidence. In FMEA, the expert’s assessment is regarded as a piece of evidence. The divergence between different assessments will be converted into uncertainty of assessment and relative weight of experts. The specific conversion will be carried out according to the following process:

Step 1: Identify potential failure modes in the target system based on past experience.

Step 2: The risk factors of these failure modes are assessed by experts, and the assessments are modeled as BPA. Assume that the ith expert’s assessments of a risk factor are modeled as a mass function $m_i=$($m(1), m(2),\dots , m(10)$), the $m(\theta )$ represent that the probability of the expert gives the level as $\theta$. m$(\theta )$ satisfy that $\sum _{\theta =1}^{10}m(\theta )=1$.

Step 3: BJS is used to measure the divergence between each expert’s assessment, and the divergence matrix (DMM) is constructed. The DMM is defined as follows:

17 $$\begin{aligned} DMM= \begin{bmatrix} BJS_{11} &{} BJS_{12} &{} \dots &{} BJS_{1n}\\ BJS_{21} &{} BJS_{22} &{} \dots &{} BJS_{2n}\\ \dots &{} \dots &{}\dots &{}\dots \\ BJS_{n1} &{} BJS_{n2} &{} \dots &{} BJS_{nn} \end{bmatrix} \end{aligned}$$

where $BJS_{ij}$ represents the divergence between $m_i$ and $m_j$. Obviously, the DMM has the following two characteristics:

1. The values on the main diagonal of DMM are 0, because when the two pieces of evidence are exactly the same, i.e., $m_1 = m_2$, BJS ($m_1 , m_2$) = 0, indicating that there is no divergence between the two pieces of evidence, which also conforms to the definition of BJS.
2. DMM is a symmetric square matrix because BJS satisfies symmetry.

Step 4: Calculate the average divergence among assessments, which is defined as follows:

18 $$\begin{aligned} \tilde{BJS_i} = \frac{\sum _{j=1}^{n}BJS_{ij}}{n-1} \qquad 1\le i \le n,1\le j \le n \end{aligned}$$

It means that summing all data in column i of DMM and dividing it by n-1. The result is the average divergence between $m_i$ and other mass functions.

Step 5: The weight of experts is defined as follows:

19 $$\begin{aligned} Wei_i=\left\{ \begin{array}{l} \frac{1}{n}, \tilde{BJS_i}=0. \\ \frac{Sup_i}{\sum _{s=1}^{n}Sup(m_s)},\tilde{BJS_i}\ne 0. \\ \end{array} \right. \end{aligned}$$

where the $Sup(m_i)$ represents the support degree,and $Sup(m_i)$ is defined as:

20 $$\begin{aligned} Sup(m_i)=\frac{1}{\tilde{BJS_i}}. \end{aligned}$$

When the $\tilde{BJS_i}=0$. It means that all of assessments are same, there is no divergence among them, so the the weights will be equally distributed. When the $\tilde{BJS_i}\ne 0$. The average divergence is converted into the degree of support, and the weight of experts is obtained by support degree weighting.

Step 6: Since the risk assessments by experts are divided into multiple levels (i.e., $m_i = (m(1),m(2),\dots ,m(10)$), the comprehensive value of risk factors needs to be calculated before calculating the RPN value. The comprehensive value of risk factors is defined as follows:

21 $$\begin{aligned} \begin{aligned} O = \sum _{j=1}^{10}\theta _j\times m(\theta _j)\\ S = \sum _{j=1}^{10}\theta _j\times m(\theta _j)\\ D = \sum _{j=1}^{10}\theta _j\times m(\theta _j) \end{aligned} \end{aligned}$$

In tradition, the expert divides his or her assessments into 10 levels, and each level corresponds to a risk value (represented by $\theta _j$ and $\theta _j\in [1,10]$). For example, an expert’s assessment of the severity (s) of a failure mode is $(m(1)=0.8, m(2)=0.1, m(3)=0.1)$, which means that 80$\%$ of people think that the failure is not serious, 10$\%$ think that the failure is moderately serious, and 10$\%$ think that the failure is very serious. Then the comprehensive value of the risk factor S is: S=0.8$\times$1+0.1$\times$2+0.1$\times$3=1.3.

Step 7: The new RPN value is calculated according to the comprehensive value of risk factors and the weighted results of expert evaluation, which is defined as follows:

22 $$\begin{aligned} BJSRPN = \frac{\sum _{i=1}^{n} O_i\times Wei(O_i)\times S_i\times Wei(S_i)\times D_i \times Wei(D_i)}{n} \end{aligned}$$

**Figure 1:**
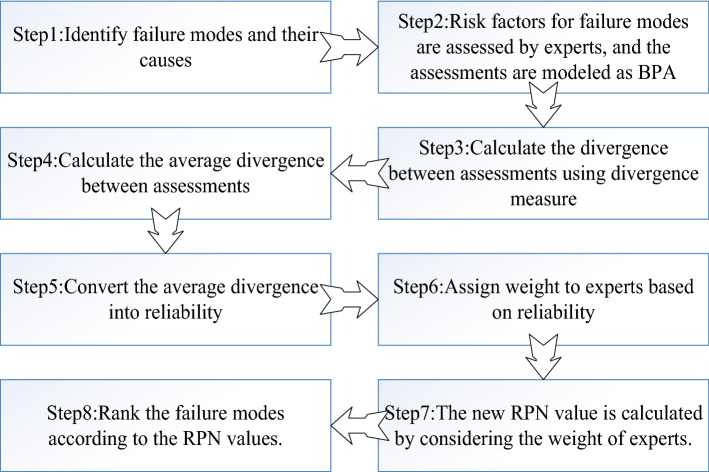
Flow chart of calculating RPN value with the proposed method.

Finally, all failure modes are ranked according to RPN values. We will know which failure modes have a higher priority and focus on them. The specific execution flow of the new method is shown in Fig. 1. It is worth noting that the weight of experts is considered in the calculation of the new RPN, and the weight is obtained by combining all assessments, not obtained independently from one piece of evidence. In other words, when the assessment of one expert changes, the weight of other experts will also be affected.

**Table 2:** The FMEA of the sheet steel production process in Guilan steel factory.

NO.	Failure mode(FM)	Cause of failure(CF)
$A_1$	Non-acceptable formation	Non-conductive scrap
$A_2$	Nipple thread pitted	Proper coverage not obtained
$A_3$	Arc formation loss	Leakage of water, proper gripping loss
$A_4$	Burn-out electrode	Cooler not working properly
$A_5$	Breaking of house of pipe	Wearing of pipe due to use
$A_6$	Problem in movement of arm	Severe leakage
$A_7$	Refractory damage	Due to slag
$A_8$	Formation of steam	Roof leak
$A_9$	Refractory line damage	By hot gas
$A_{10}$	Movement of roof stop	Jam of plunger in un loader valve

**Table 3:** The belief structure of the first failure mode.

Experts	Occurrence(O)	Severity (S)	Detection(D)
Expert1	m(1)=0.1 m(2)=0.2 m(3)=0.7	m(1)=0.8 m(2)=0.1 m(3)=0.1	m(1)=0.2 m(2)=0.5 m(3)=0.3
Expert2	m(2)=0.4 m(3)=0.6	m(1)=0.7 m(3)=0.3	m(1)=0.3 m(2)=0.4 m(3)=0.3
Expert3	m(1)=0.1 m(2)=0.4 m(3)=0.5	m(1)=0.8 m(2)=0.2	m(1)=0.2 m(2)=0.5 m(3)=0.3

**Table 4:** The comprehensive value of risk factors of $FM_1$.

$FM_1$	O	S	D
Expert1	$O_1$=2.6	$S_1$=1.3	$D_1$=2.1
Expert2	$O_2$=2.6	$S_2$=1.6	$D_2$=2.0
Expert3	$O_3$=2.4	$S_3$=1.2	$D_3$=2.1

**Table 5:** The average divergence of risk factors in $FM_1$.

$FM_1$	O	S	D
Expert1	BJS($O_1$)=0.0569	BJS($S_1$)=0.0762	BJS($D_1$)=0.0056
Expert2	BJS($O_2$)=0.0653	BJS($S_2$)=0.1713	BJS($D_2$)=0.0113
Expert3	BJS($O_3$)=0.0449	BJS($S_3$)=0.1573	BJS($D_3$)=0.0056

**Table 6:** The support degree of risk factors in $FM_1$.

$FM_1$	O	S	D
Expert1	Sup($O_1$)=17.5626	Sup($S_1$)=13.1228	Sup($D_1$)=177.3345
Expert2	Sup($O_2$)=15.3172	Sup($S_2$)=5.8384	Sup($D_2$)=88.6672
Expert3	Sup($O_3$)=22.2535	Sup($S_3$)=6.3560	Sup($D_3$)=177.33451

## Applications and discussion

### Application 1

#### Experiment process

To verify the feasibility of the new method in this work, the application example in^52^ was referenced to conduct an experiment in this work, and the experimental results are compared with the other four methods. In the end, the effectiveness of this method has been verified. The experimental steps are as follows:

1. Find all the failure modes in the target system. As shown in Table 2, this is an application example of a steel plate production process with 10 failure modes.

2. Collect those assessments of the risk factor from experts. Taking the first failure mode as an example, the assessment results are shown in Table 3 (the rest of the assessment results can be found in^52^). Three experts assessed the risk factors, and these assessments were divided into 3 levels, from which the comprehensive value of risk factors can be calculated by formula 21, and the result is shown in Table 4.

3. Calculate the divergence between two assessments using formula 12, and structure the divergence matrix using formula 17. In $FM_1$, the divergence matrix was structured as follows according to the values in Table 3.
  $$\begin{aligned} DMM(O)= & {} \begin{bmatrix} 0 &{}0.0773 &{}0.0366\\ 0.0773 &{}0 &{}0.0533\\ 0.0366 &{}0.0533 &{}0 \end{bmatrix} \\ DMM(S)= & {} \begin{bmatrix} 0 &{}0.0902 &{}0.0623\\ 0.0902 &{}0 &{}0.2524\\ 0.0623 &{}0.2524 &{}0 \end{bmatrix} \\ DMM(D)= & {} \begin{bmatrix} 0 &{}0.0113 &{}0\\ 0.0113 &{}0 &{}0.0113\\ 0 &{}0.0113 &{}0 \end{bmatrix} \end{aligned}$$
4. Using formulas 18 and 20 to calculate the average divergence and the support degree between assessments, the results are shown in Tables 5 and 6.

5. Using formula 19 to calculate the weight of experts, as shown in Table 7.

6. Using formula 22 to calculate the RPN value of $FM_1$ in combination with the data in Table 4 and Table 7, the result is 0.2735. Repeat all the above steps to calculate the RPN value of other FMs. RPN values and the ranking result according to RPN values are shown in Table 8. The ranking result is $FM_4> FM_7> FM_3> FM_8> FM_1> FM_{10}> FM_2> FM_5> FM_6 > FM_9$. Because $FM_4$ is ranked first, in practice, the managers should pay more attention to the monitoring and management of $FM_4$, followed by $FM_7$. $FM_9$ has the lowest RPN value, ranks last, and will be given the least attention. In addition, it should be noted that for the two groups of failure modes with very close or even the same RPN values, such as $FM_5$ and $FM_6$, although they have the sequence based on RPN, they should be given the same attention as much as possible.

**Table 7:** The support degree of risk factors in $FM_1$.

$FM_1$	O	S	D
Expert1	Wei($O_1$)=0.3185	Wei($S_1$)=0.5183	Wei($D_1$)=0.4000
Expert2	Wei($O_2$)=0.2778	Wei($S_2$)=0.2306	Wei($D_2$)=0.2000
Expert3	Wei($O_3$)=0.4036	Wei($S_3$)=0.2511	Wei($D_3$)=0.4000

**Table 8:** RPN values and the ranking result.

Item	$FM_1$	$FM_2$	$FM_3$	$FM_4$	$FM_5$	$FM_6$	$FM_7$	$FM_8$	$FM_9$	$FM_{10}$
RPN	0.2735	0.2094	0.2948	0.4895	0.1969	0.1969	0.3642	0.2948	0.1969	0.2503
Rank	5	7	3	1	8	9	2	4	10	6

#### Experimental result of application 1

**Figure 2:**
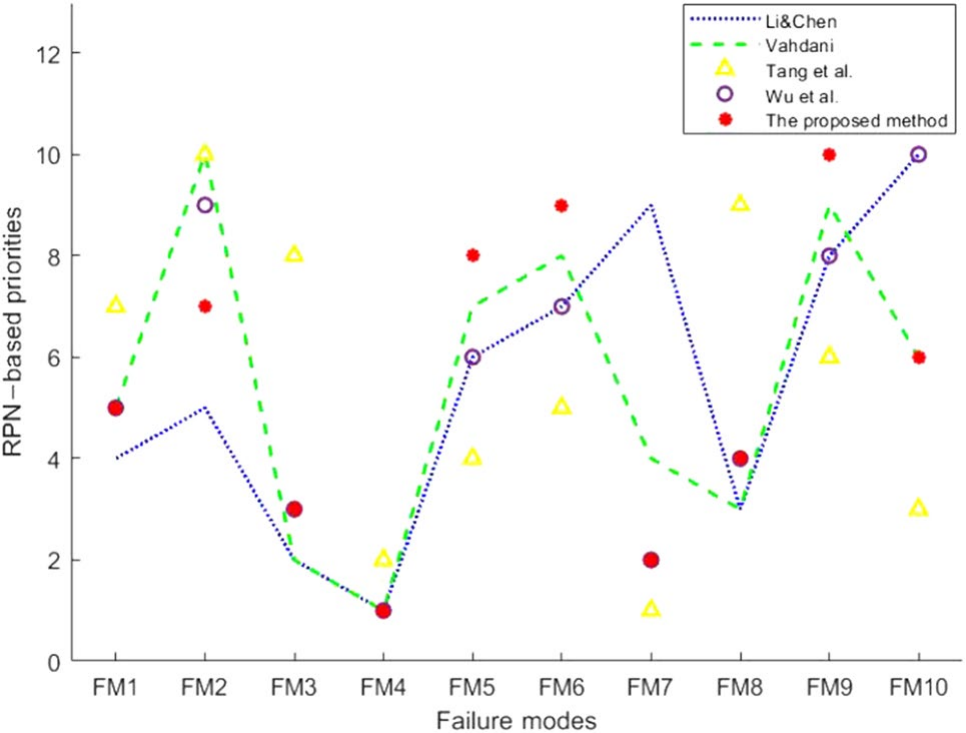
Ranking of failure modes with different methods.

In order to verify the correctness of the method proposed in this work, the experimental results are compared with the results in papers^27,28,52,53^. In^52^, Li and Chen used the grey correlation projection method to deal with the uncertainty between expert assessments. In^53^, Vahdani et al. combined the fuzzy belief TOPSIS method with FMEA to improve the traditional FMEA model. The correctness of the other methods has been well verified in their articles. The comparison results between the method proposed in this work and the other methods are shown in Fig. 2.

**Figure 3:**
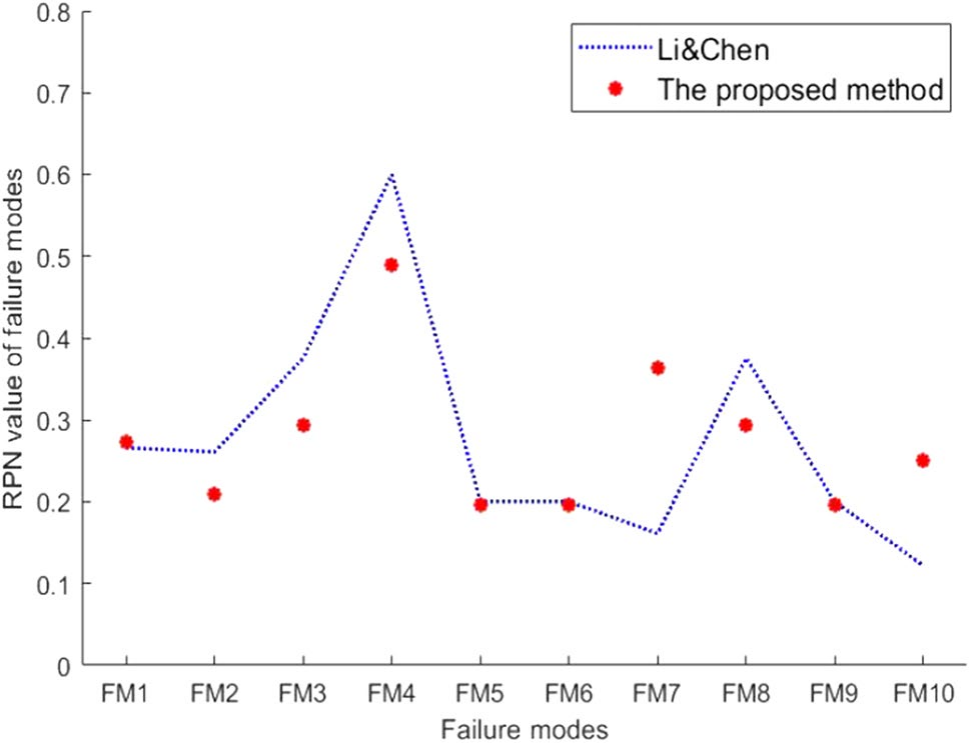
RPN values of Li and Chen’s method and the proposed method.

It shows that the ranking result obtained by this method has the same trend as those obtained by the other methods (that is, the relative position of ranking between failure modes does not change much), especially the $FM_4$ ranked first, which is completely consistent with the results of other three methods, which ensure that in the practical application, focus is on the failure mode with the highest risk initially. The results indicate there is a certain amount of distinction through different methods. We considered that this distinction may be caused by the RPN value, so we compared the RPN values with Li and Chen’s method. The results are shown in Fig. 3. It indicates that the RPN values produced by Li and Chen’s method and the proposed method are very close.

### Application 2

#### Experiment process

In order to better verify the application of this method in FMEA, we used another example in^54^ to verify it. There are 17 failure modes in this example, and the data was processed more accurately in^48^. Some of the assessments are shown in Table 9.

**Table 9:** belief structure of the first failure mode in application two.

Risk	Expert1	Expert2	Expert3
O	m(3)=0.4 m(4)=0.6	m(3)=0.9 m(4)=0.1	m(3)=0.8 m(4)=0.2
S	m(6)=0.1 m(7)=0.8 m(8)=0.1	m(6)=0.1 m(7)=0.8 m(8)=0.1	m(6)=0.1 m(7)=0.8 m(8)=0.1
D	m(1)=0.1 m(2)=0.8 m(3)=0.1	m(1)=0.1 m(2)=0.8 m(3)=0.1	m(1)=0.1 m(2)=0.8 m(3)=0.1

The calculation is similar to the application one, due to space, the calculation process will not be described here. Table 10 shows the RPN values and the ranking result. The ranking result is $FM_9>FM_2> FM_{14}> FM_6> FM_{10}> FM_{12}> FM_{11}> FM_{13}> FM_1> FM_{15}> FM_{17}> FM_3> FM_7> FM_{16}> FM_4> FM_8 > FM_5$. The result is consistent with the preliminary assessment.

**Table 10:** The RPN values and ranking result.

Item	$FM_1$	$FM_2$	$FM_3$	$FM_4$	$FM_5$	$FM_6$	$FM_7$	$FM_8$	$FM_9$
RPN values	1.6858	2.3881	1.1111	0.6293	0.1554	2.2222	0.7778	0.5896	2.8293
ranking	9	2	12	15	17	4	13	16	1
Item	$FM_{10}$	$FM_{11}$	$FM_{12}$	$FM_{13}$	$FM_{14}$	$FM_{15}$	$FM_{16}$	$FM_{17}$	
RPN values	2.2222	1.8519	2.0279	1.8370	2.2370	1.5280	0.6983	1.2224	
ranking	5	7	6	8	3	10	14	11	

#### Experimental result of application 2

**Figure 4:**
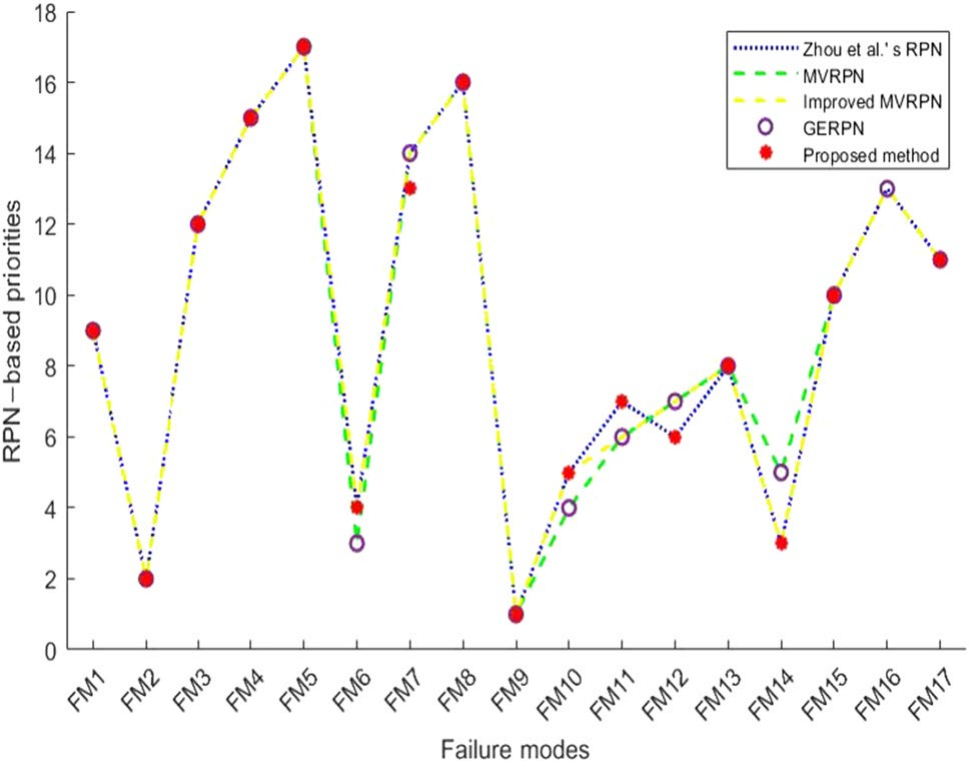
Ranking of failure modes with different methods.

The comparison of the ranking result with other methods( MVRPN^54^, Improved MVRPN^48^, GERPN^55^, Zhou et al.’s method^56^) is shown in Fig. 4. The ranking result is very close to the other methods, especially exactly the same as Zhou et al.’s method, which makes the usability of the method further verified. As for the comparison of the RPN values, it is shown in Table 11. The RPN values of this method are generally smaller than other RPN values. In case where all the assessments are different, other methods produces 5 same RPN values ($FM_6$ and $FM_{10}$, $FM_{11}$, $FM_{12}$ and $FM_{13}$), and this method produces only 2 same RPN values($FM_6$ and $FM_{10}$). The reason for this gap is the way experts are assigned weight.

**Table 11:** A comparison of RPN values.

Item	Zhou et al.’s RPN	MVRPN	Improved MVRPN	GERPN	Proposed method
$FM_1$	46.4875	42.56	42.56	3.4910	1.6858
$FM_2$	64.7921	64.00	64.05	3.9994	2.3881
$FM_3$	30.0000	30.00	30.00	3.1069	1.1111
$FM_4$	17.5822	18.00	17.97	2.6205	0.6293
$FM_5$	3.6671	4.17	3.14	1.6095	0.1554
$FM_6$	60.0000	60.00	60.00	3.9143	2.2222
$FM_7$	21.0000	21.00	21.00	2.7586	0.7778
$FM_8$	16.2000	15.00	15.00	2.4660	0.5896
$FM_9$	70.5947	78.92	79.57	4.2881	2.8293
$FM_{10}$	60.0000	60.00	60.00	3.9143	2.2222
$FM_{11}$	50.0000	50.00	50.00	3.6836	1.8519
$FM_{12}$	53.8039	50.00	50.00	3.6836	2.0279
$FM_{13}$	49.3333	50.00	50.00	3.6836	1.8370
$FM_{14}$	60.6337	60.00	60.04	3.9143	2.2370
$FM_{15}$	41.9161	42.00	42.09	3.4756	1.5280
$FM_{16}$	21.2967	23.88	23.86	2.8794	0.6983
$FM_{17}$	31.2810	30.05	30.05	3.1089	1.2224

### Discussion

In general, the feasibility of the new method is verified by the above cases. One characteristic of this method is that the RPN value generated is small, but it does not affect the final sorting result. Compared with other methods, the new method is less likely to produce the same RPN values, which can better overcome the defects of the traditional FMEA and make the evaluation more accurate. In addition, this method also has some issues that need to be improved. The uncertainty between assessments of the same risk factor can represent the weights of the experts, but the uncertainty between assessments of different risk factors cannot represent the weights of different risk factors. Other uncertainty measures can be introduced into this method to measure the weight between different risk factors.

## Conclusion

The uncertainty of expert assessment has always been an inevitable problem in risk management. Due to the effectiveness of FMEA in risk assessment, managers pay more and more attention to the accuracy of FMEA in failure mode assessment to ensure the safe operation of the target system. Therefore, the traditional FMEA has great limitations. At the same time, effective methods are also needed to improve the problems of the traditional FMEA.

This work proposed a method based on the divergence measure to deal with the uncertainty of expert assessment. This method transforms the uncertainty of experts’ subjective assessment into experts’ weight, and attempts to improve the accuracy of assessment from the perspective of experts’ weight. At the same time, the divergence measure highlights the correlation between assessments, so that the assessments are no longer isolated. Finally, a case of a steel plate production process is used to verify the practicability of this method, and excellent results are obtained.

The core idea in this work is that by using the divergence measure to obtain the divergence between assessments and converting this divergence into the support degree of assessments, the support degree will represent the weight of experts. In the following research, we can apply this method to other fields to deal with the uncertainty of subjective assessments and consider introducing information entropy to measure the quantity of information in assessments to improve this method from the perspective of the weighted risk factor. In addition, the fusion of different pieces of evidence with potential conflict has always been an open issue in the Dempster-Shafer evidence theory. Thus, we can improve this method and apply it to the fusion of conflicting assessments.

## Data Availability

All data generated or analysed during this study are included in this published article.
